# Novel lumbar plexus block versus femoral nerve block for analgesia and motor recovery after total knee arthroplasty

**DOI:** 10.1515/med-2023-0881

**Published:** 2024-01-12

**Authors:** Wen-Yi Gong, Feng Zou, Xiao-Fang Yue, Chen-Guang Li, Jing-Yu Zhang, Kun Fan

**Affiliations:** Department of Anesthesiology, Zhongshan Wusong Hospital Affiliated to Fudan University, Shanghai, China; Department of Anesthesiology, Shanghai Sixth People’s Hospital Affiliated to Shanghai Jiaotong University School of Medicine, Shanghai, 200233, China; Department of Neurology, Shanghai Sixth People’s Hospital Affiliated to Shanghai Jiaotong University School of Medicine, Shanghai, China; Department of Anesthesiology, Tianshui First People’s Hospital, Tianshui, Gansu, China; Department of Anesthesiology, The Second Hospital, Lanzhou University, Lanzhou, Gansu, China

**Keywords:** total knee arthroplasty, lumber plexus block, femoral nerve block, ultrasound

## Abstract

This study aimed to compare the postoperative analgesic efficacy and motor recovery of a novel lumbar plexus block (LPB) with that of a femoral nerve block (FNB) after total knee arthroplasty (TKA). Forty patients who underwent TKA were randomised equally into an lumbar plexus and sciatic nerve (LS) group (receiving novel LPB) and an femoral and sciatic nerves (FS) group (receiving FNB). The assessed variables were the onset time of pain, time to the first analgesic request, pain scores, motor block at 6, 12, and 24 h after TKA, and the number of patients receiving successful blockade for each branch of the lumbar plexus. In the LS group, the femoral, lateral femoral cutaneous, genitofemoral, iliohypogastric, ilioinguinal, and obturator nerves were blocked in 18, 20, 16, 18, 15, and 19 patients. Compared to the FS group, the LS group had a significantly shorter onset time of pain and time to the first analgesic request, a significantly larger total postoperative dose of sufentanil, significantly higher numeric rating scale scores for both rest and dynamic pain at 6, 12, and 24 h, and faster motor recovery. Novel ultrasound-guided LPB has a high blocking success rate and provides inferior postoperative analgesia, but faster motor recovery after TKA than FNB.

## 1 Introduction

Total knee arthroplasty (TKA) is a common orthopaedic procedure known to cause moderate-to-severe postoperative pain [1,2], which can significantly affect patient outcomes. Inadequate pain management may result in acute adverse effects such as immunosuppression and reduced mobility, leading to deep vein thrombosis (DVT), pulmonary embolism, myocardial infarction, and pneumonia [3]. Long-term consequences include chronic pain and opioid dependence due to prolonged narcotic consumption [3,4]. Despite the numerous analgesic options available for knee surgery, approximately 50% of TKA patients still experience postoperative pain [5], and 20% remain dissatisfied owing to suboptimal pain relief [6].

Peripheral nerve blocks, especially the femoral nerve block (FNB), play a crucial role in minimising postoperative pain after TKA [7]. A combination of FNB and sciatic nerve block (SNB) has been shown to decrease pain intensity and additional analgesic requirements [8–10]. Sensory innervation around the knee joint is mainly from the femoral nerve (FN), obturator nerve (ON), lateral femoral cutaneous nerve, and sciatic nerve (SN) [11], which suggests a potential advantage of the lumbar plexus block (LPB) over FNB. However, its efficacy compared with that of FNB for TKA remains controversial. Some studies have proposed that a combined psoas-sciatic block is more effective than an FNB-sciatic block [12], whereas others have shown no significant difference between LPB and FNB in terms of pain relief after TKA [13].

Most ultrasound-guided techniques for LPB require a lateral decubitus position, which can be inconvenient and painful for patients [14]. Recently, a new short-axis ultrasound-guided LPB approach in the supine position was developed, which is considered safe, effective, and comfort for patients undergoing TKA. This study aimed to investigate the efficacy and safety of this novel technique compared with FNB and to evaluate its potential utility in perioperative pain management.

## 2 Materials and methods

### 2.1 Study design

This prospective, single-centre, randomised clinical trial was conducted in accordance with the ethical principles of the Helsinki Declaration and was approved by the Medical Ethics Committee of Zhongshan Wusong Hospital Affiliated to Fudan University, Shanghai, China (approval no. 2020-Y-19, approval date 15 June 2020). This study was registered in the Chinese Clinical Trial Registry (Registration No. ChiCTR2100048454).

### 2.2 Patients

The trial was performed in the operating room of the Zhongshan Wusong Hospital Affiliated to Fudan University, between July 2020 and March 2021. The study enroled 40 patients, aged 18 years or older, who underwent TKA for osteoarthritis and had an American Society of Anaesthesiologists physical status classification (ASA) of Ⅰ–Ⅲ. Written informed consent was obtained from all the participants and their relatives. The exclusion criteria were as follows: psychiatric disorders, inability to cooperate or communicate in Chinese, chronic pain, ingestion of any pain medication, lower limb neuropathy, allergy to nonsteroidal anti-inflammatory drugs, renal failure, myocardial ischaemia, cerebral infarction, liver injury, active gastric ulcer, and contraindications to regional anaesthesia (coagulopathy, local infection at the block site, and/or allergy to a local anaesthetic).

### 2.3 Randomisation and blinding

Patients were randomised into two groups using a computer-generated randomisation table (http://www.random.org) by a researcher who was not involved in the study, in a 1:1 allocation ratio. The lumbar plexus and sciatic nerve (LS) group received a lumbar plexus (LP)–SN block, whereas the femoral and sciatic nerves (FS) group received an FN–SN block. Before the arrival of the patient in the operating room, the anaesthetist opened a sequentially numbered, sealed, opaque envelope containing the patient’s group assignment. The patients, research coordinator, statisticians, surgeons, and intraoperative anaesthesiologists were blinded to the group assignments.

### 2.4 Preparation for nerve blocks

All patients underwent standard monitoring, including heart rate, non-invasive blood pressure, oxygen saturation, skin temperature, and bispectral index upon arrival in the operating room. Intravenous access was established in the forearm of each patient. Before the nerve block procedure, patients were given 40 mg of intravenous parecoxib and 1 μg/kg of intravenous dexmedetomidine administered over a period of 10 min.

### 2.5 Nerve blocks

#### 2.5.1 LS group

**Figure 1:**
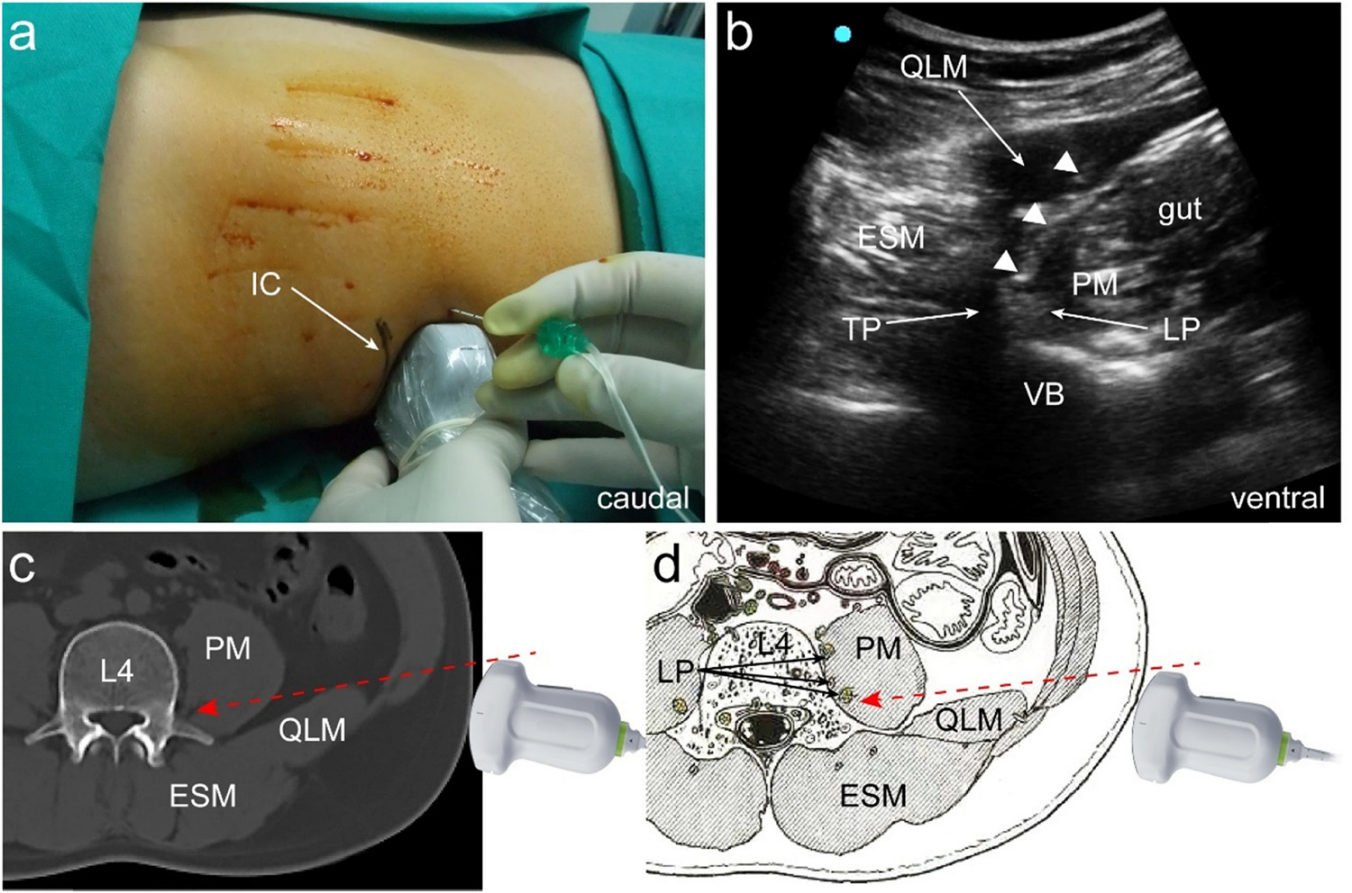
Ultrasound-guided lumber plexus block with short axis in supine position: (a) probe position, (b) ultrasound-guided lumber plexus block in supine position at L4 level, (c) schematic computed tomography diagram of ultrasound-guided lumber plexus block in supine position, and (d) schematic anatomical illustration of ultrasound-guided lumber plexus block in supine position. IC, iliac crest; PM, psoas major muscle; QLM, quadratus lumborum muscle; ESM, erector spinae muscle; VB, vertebral body; TP, transverse process; LP, lumber plexus. Three white triangles point at the trajectory of a needle. Red dotted arrows denote the trajectory of a needle.

The patient was positioned in a supine posture with the legs in a naturally straight alignment. Ultrasound imaging was performed using a 2–5 MHz convex array probe (SonoSite SⅡ; Fujifilm Sonosite, Inc., Bothell, WA, USA) placed in the transverse plane at the posterior axillary line near the iliac crest (Figure 1a). The quadratus lumborum, erector spinae, psoas major, L4 vertebral body, and L4 transverse process were identified using ultrasonography (Figure 1b–d). The LP was detected as a short-axis hyperechoic structure located in frontal proximity to the L4 transverse process and within the posterior one-third of the psoas major muscle (Figure 1b–d). A 22-gauge puncture needle was inserted in-plane from the ventral side of the probe (Figure 1b–d). Following puncture needle penetration through the quadratus lumborum into the psoas major muscle, aspiration was performed to ensure that there were no vascular punctures. Finally, 30 mL of 0.33% ropivacaine was added adjacent to the LP.

Ultrasound-guided SNB was performed in the upper medial thigh with the patient in the supine position, following a standard procedure outlined in the literature [15]. Twenty millilitres of 0.33% ropivacaine was injected.

#### 2.5.2 FS group

In accordance with previous studies [16,17], ultrasound-guided FNB was performed in the inguinal region by injecting 30 mL of 0.33% ropivacaine with the patient in the supine position. Ultrasound-guided SNB was performed as described above, using 20 mL of 0.33% ropivacaine.

### 2.6 Intraoperative management

The anaesthetic procedure used a laryngeal mask for general anaesthesia and was performed by an anaesthetist who was not involved in the nerve block manipulation. Propofol at a dose of 3 mg/kg and sufentanil at 0.2 µg/kg were administered for induction of anaesthesia. Sevoflurane was used to maintain anaesthesia, with titration of the inhaled concentration to maintain a bispectral index of 40–60. Intraoperatively, a 5 µg bolus of sufentanil was administered if the baseline systolic blood pressure or heart rate increased by 20%. Ondansetron (4 mg) was administered intravenously for prophylaxis of postoperative nausea and vomiting (PONV) 30 min prior to surgery. Postoperatively, patients received patient-controlled intravenous analgesia (PCIA) for rescue analgesia. The PCIA device dispensed 2 µg bolus doses of sufentanil with a 10 min lock-out interval, without a basal infusion.

### 2.7 Postoperative management

The patients were transferred to the post-anaesthesia care unit after surgery and were shifted to the surgical ward only when the Steward score was ≥4 after removal of the laryngeal mask. Intravenous parecoxib was administered at a dose of 40 mg at 12 h and 24 h after surgery. Pain was evaluated using a numerical rating scale (NRS) score that ranges from 0 (no pain) to 10 (worst imaginable pain). PCIA was initiated if the NRS score was ≥4 or the pain was intolerable. Patients with PONV were treated with 10 mg intravenous metoclopramide.

### 2.8 Outcome measures

The primary outcome was the time of onset of postoperative pain, which was recorded by the research coordinator.

Demographic details including sex, age, height, weight, affected side, and American Society of Anaesthesiologists grade were recorded by the research coordinator for each patient. Other recorded parameters included the occurrence of PONV, dose of metoclopramide, duration of surgery, complications related to nerve block (vascular injury, haematoma, and local anaesthetic toxicity), and epidural extension (defined as either bilateral sensory block achieved to L1–L2 levels or complete motor block in both lower limbs detected 30 min after the nerve block [18]).

Nerve visibility was assessed by the anaesthetist performing the nerve block using a 4-point scale, in which 0 indicated that the nerve was not visible, 1 indicated that the nerve was barely visible, 2 indicated that the nerve was clearly visible, and 3 indicated that the nerve was very clearly visible [19]. Patients with a visibility score of 0 for nerves requiring blockade were excluded from the study. The onset time of the sensory blockade was recorded by the research coordinator. Thirty minutes after the nerve block, the research coordinator performed a sensory examination in the regions innervated by the LP branches (the iliohypogastric, ilioinguinal, genitofemoral, obturator, lateral femoral cutaneous, and FNs) [20], using pinprick sensation scores, where 0 indicated no sensation loss, 1 indicated partial sensation loss, and 2 indicated complete sensation loss. The research coordinator recorded the total intraoperative sufentanil dose.

The time to first analgesic request, sufentanil consumption within 24 h postoperatively, NRS scores for rest pain and dynamic pain (pain in the operated knee on active or passive flexion to 45° [21]) at 6, 12, and 24 h postoperatively, Bromage score (0  =  free movement of the legs and feet, 1  =  just able to flex the knees with free movement of the feet; 2  =  unable to flex the knees, free movement of the feet, and 3  =  unable to move the legs or feet [22]) of the affected lower limb at 6, 12, and 24 h postoperatively, and adverse effects of opioids (respiratory depression and pruritus) were also recorded by a research coordinator. Patients were followed-up by telephone 2 weeks postoperatively to assess the presence of haematuria, haematochezia, and peripheral nerve injury (sensory or motor dysfunction in the affected extremity).

### 2.9 Statistical analysis

The power calculation was based on the time of pain onset. According to our pilot study results, we assumed that the onset times of pain for patients in the LS group and FS group would be 331.78 ± 309.683 and 1225.91 ± 335.215 min, respectively. Detection of this intergroup difference with *α* = 0.05 and a power of 80% would require a sample size of 17 patients in each group. To allow for dropouts, 20 patients were allocated to the LS group and 20 patients were allocated to the FS group.

All statistical analyses were performed with IBM SPSS Statistics 22 (IBM Corp., Armonk, NY, USA) software. Data were evaluated for normality using the Shapiro–Wilk test. Normally distributed data, including age, weight, height, duration of surgery, and time to first analgesic request, are expressed as the mean (standard error of the mean). Intergroup differences were assessed for significance using the Student’s *t*-test. Non-normally distributed data, including the total dose of metoclopramide, onset time of sensory blockade, total intraoperative dose of sufentanil, onset time of pain, sufentanil consumption within 24 h postoperatively, NRS scores for rest pain and dynamic pain at 6, 12, and 24 h postoperatively, and Bromage scores at 6, 12, and 24 h postoperatively, were expressed as median (range). Intergroup differences were assessed for significance using the Mann–Whitney *U* test. Categorical variables, including sex, side, ASA score, PONV, visibility, pinprick sensation score 30 min after injection, rest pain score <4 within 24 h, and dynamic pain score <4 within 24 h are expressed as numbers. Intergroup differences were assessed using Pearson’s *χ*² test or Fisher’s exact test as appropriate. Statistical significance was set at *P* < 0.05.

## 3 Results

**Figure 2:**
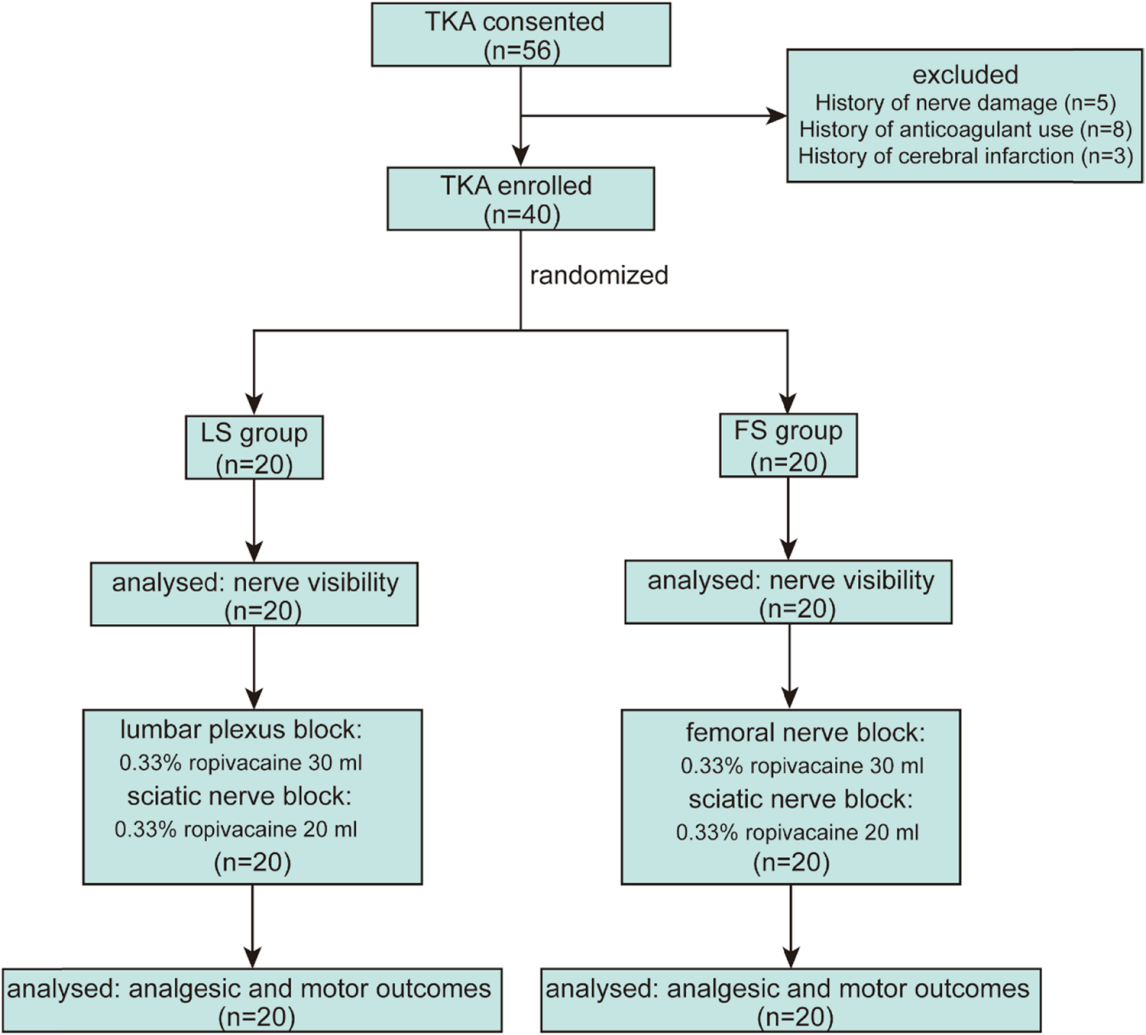
Consolidated standards of reporting trials flow diagram. LS, lumbar plexus block combined with sciatic nerve block group; FS, femoral nerve block combined with sciatic nerve block group; TKA, total knee arthroplasty.

A flowchart of the investigation is shown in Figure 2. Forty patients were enroled and randomly divided into two groups (LS or FS) through randomised grouping. No patients withdrew from the study. Their demographic data are presented in Table 1. None of the patients in either group experienced vascular injury, haematoma, local anaesthetic intoxication, epidural extension, haematuria, haematochezia, nerve injury, respiratory depression, or pruritus.

**Table 1:** Demographic characteristics, PONV, total dose of metoclopramide, and duration of surgery

Parameter	LS (*n* = 20)	FS (*n* = 20)	*P* value
Age, years	63.9 ± 1.6	65.8 ± 1.4	0.386^a^
Sex (male/female), *n*	5/15	4/16	1.000^d^
Weight, kg	66.6 ± 2.2	67.4 ± 2.4	0.871^a^
Height, cm	159.1 ± 1.7	157.4 ± 1.3	0.413^a^
Side (left/right), *n*	12/8	11/9	0.749^c^
ASA (Ⅰ/Ⅱ), *n*	3/17	0/20	0.231^d^
PONV (yes/no), *n*	5/15	7/13	0.490^c^
Total dose of metoclopramide, mg	0.0 (0.0–20.0)	0.0 (0.0–20.0)	0.545^b^
Duration of surgery, min	80.5 ± 4.3	72.0 ± 3.2	0.122^a^

Data were presented as mean ± SEM, median (range), or number of patients. ^a^Student’s *t*-test; ^b^Mann–Whitney *U* test; ^c^Pearson’s *χ*² test; ^d^Fisher’s exact test. LS, lumbar plexus block combined with sciatic nerve block group; FS, femoral nerve block combined with sciatic nerve block group; ASA, American Society of Anesthesiologists physical status; PONV, postoperative nausea and vomiting.

In the LS group, the LP was visualised in all patients, while in the FS group, the FN was visible in all patients (Table 2). In the LS group, the innervated areas of the femoral, lateral femoral cutaneous, genitofemoral, iliohypogastric, ilioinguinal, and ONs had pinprick sensation scores of 1 or 2 in 18, 20, 16, 18, 15, and 19 patients, respectively, at 30 min post-injection (Table 2). There were significantly fewer patients with a pinprick sensation score of 0 in the innervation areas of the lateral femoral cutaneous, genitofemoral, iliohypogastric, and ilioinguinal nerves in the LS group than in the FS group at 30 min post-injection (Table 2).

**Table 2:** Nerve visibility, sensory block characteristics 30 min after injection, and total intraoperative dose of sufentanil

Parameter	LS (*n* = 20)	FS (*n* = 20)	*P* value
Visibility (0/1/2/3), *n*	0/5/13/2	0/0/9/11	
Onset time of sensorial blockade, s	60.0 (30.0–110.0)	50.0 (30.0–90.0)	0.354^b^
**Pinprick sensation score 30 min after injection,** ***n***			
**FN**			
0	2	0	0.487^d^
1	5	2	0.407^d^
2	13	18	0.127^d^
**Lateral femoral cutaneous nerve**			
0	0	13	0.000^c^
1	6	6	1.000^c^
2	14	1	0.000^c^
**Genitalfemoral nerve**			
0	4	20	0.000^c^
1	5	0	0.047^d^
2	11	0	0.000^c^
**Iliohypogastric nerve**			
0	2	20	0.000^c^
1	9	0	0.001^d^
2	9	0	0.001^d^
**Ilioinguinal nerve**			
0	5	20	0.000^c^
1	5	0	0.047^d^
2	10	0	0.000^c^
**ON**			
0	1	2	1.000^d^
1	12	13	0.744^c^
2	7	5	0.490^c^
Total intraoperative dose of sufentanil, μg	10.0 (5.0–25.0)	10.0 (10.0–30.0)	0.052^b^

Data were presented as median (range), or number of patients. ^b^Mann–Whitney *U* test; ^c^Pearson’s *χ*² test; ^d^Fisher’s exact test. LS, lumbar plexus block combined with sciatic nerve block group; FS, femoral nerve block combined with sciatic nerve block group.

The onset time of pain and time to the first analgesic request in the LS group were significantly shorter when compared to the FS group (Table 3). The total dose of sufentanil at 0–6, 6–12, and 12–24 h postoperatively was significantly greater in the LS group than in the FS group (Table 3). The NRS scores for both rest and dynamic pain were significantly higher in the LS group than in the FS group at 6, 12, and 24 h postoperatively (Table 3). The LS group had fewer patients with rest and dynamic pain scores less than four within 24 h postoperatively than the FS group (Table 3). Bromage scores at 12 and 24 h were significantly lower in the LS group than in the FS group (Table 3).

**Table 3:** Postoperative sensory and motor block characteristics

Parameter	LS (*n* = 20)	FS (*n* = 20)	*P* value
Onset time of pain, min	437.5 (70.0–1150.0)	1270.0 (700.0–1605.0)	0.000^b^
Time to first analgesic request, min	609.0 ± 87.4	1347.5 ± 62.8	0.000^a^
**Total postoperative dose of sufentanil, μg**			
0–6 h	0.0 (0.0–26.0)	0.0 (0.0–0.0)	0.004^b^
6–12 h	12.0 (0.0–26.0)	0.0 (0.0–2.0)	0.000^b^
12–24 h	9.0 (2.0–20.0)	3.0 (0.0–14.0)	0.001^b^
**NRS score of rest pain**			
6 h	0.0 (0.0–5.0)	0.0 (0.0–0.0)	0.002^b^
12 h	3.0 (0.0–5.0)	0.0 (0.0–2.0)	0.000^b^
24 h	3.0 (1.0–4.0)	1.0 (0.0–3.0)	0.001^b^
Rest pain score <4 within 24 h, *n*	12	20	0.003^d^
**NRS score of dynamic pain**			
6 h	1.0 (0.0–7.0)	0.0 (0.0–0.0)	0.000^b^
12 h	4.0 (0.0–7.0)	0.0 (0.0–4.0)	0.000^b^
24 h	4.0 (2.0–7.0)	2.5 (0.0–6.0)	0.000^b^
Dynamic pain score <4 within 24 h, *n*	2	15	0.000^c^
**Bromage score**			
6 h	1.0 (0.0–3.0)	2.0 (1.0–2.0)	0.184^b^
12 h	0.0 (0.0–1.0)	1.0 (0.0–2.0)	0.000^b^
24 h	0.0 (0.0–0.0)	0.0 (0.0–2.0)	0.001^b^

Data were presented as mean ± SEM, median (range), or number of patients. ^a^Student’s *t*-test; ^b^Mann–Whitney *U* test; ^c^Pearson’s *χ*² test; ^d^Fisher’s exact test. LS, lumbar plexus block combined with sciatic nerve block group; FS, femoral nerve block combined with sciatic nerve block group.

## 4 Discussion

In this study, successful ultrasound identification of the LP was achieved in all participants, and the LP branches were blocked with a success rate ranging from 75 to 100%. Various ultrasound-guided LPB techniques have been described, including the paramedian transverse, median transverse, shamrock, and trident approaches. Previous studies have demonstrated that the LP is often identifiable under ultrasound using the trident approach [23], the median transverse approach achieves an identification success rate of 100% [24], the paramedian transverse approach obtains identification rates of 57 and 67% [19,25], and the shamrock approach reveals the LP in 89.1% and almost 100% of cases [23,26]. The blocking success rate of LP branches was reported to be 80–100% using the paramedian transverse approach, 70–90% using the Shamrock approach, and 80–100% using the median transverse approach [14]. Thus, our novel technique offers LP visibility and LPB success rates comparable to those of the traditional LPB methods.

Our findings revealed that within the first 24 h after TKA, the postoperative analgesic efficacy of LPB was suboptimal compared to that of FNB. Anatomically, the sensory nerves of the knee primarily originate from the femoral, obturator, and tibial nerves [27]. A network meta-analysis indicated that FNB and the adductor canal block are crucial for postoperative analgesia following TKA [24], while the use of the ON block for postoperative analgesia after TKA is still debatable [13,28]. Our results indicated that the analgesic duration of the femoral–sciatic nerve block was up to 22 h, which further supports the importance of blocking both the FN and SNs for optimal pain management after TKA. Although some studies suggest that LPB simultaneously blocks the FN and ON, thereby achieving superior postoperative analgesia after TKA compared to FNB, it is only effective in continuous blocks [12]. Our findings illustrate that a single-injection LPB was able to effectively block the ON and FN; however, the duration of blocking may potentially be shorter, resulting in poorer analgesic effects after TKA than single-injection FNB. In conclusion, the femoral‒sciatic nerve block offers superior analgesia compared to the LP–SN block when a single-injection nerve block is performed to manage postoperative pain after TKA.

In this study, it was found that despite the majority of patients in the FS group having an NRS score less than 4 for rest pain and receiving minimal amounts of opioids, the group demonstrated higher Bromage scores at both 12 and 24 h postoperatively than the LS group. This indicated that FNB had a greater impact on lower extremity mobilisation. It is important to note that peripheral nerve blocks can result in decreased muscle strength, leading to an increased risk of falls and difficulty with ambulation after a TKA. This may impede early functional exercise and potentially increase postoperative nociceptive adverse events [29]. Additionally, without traditional anticoagulation therapy following TKA, 40–62% of patients are likely to develop postoperative DVT [30]. The addition of combined FNB and SNB can further delay lower-extremity mobilisation, elevating the risk of postoperative DVT. Considering these risks, the LPB approach is a superior alternative to FNB for TKA patients at high risk of falls or DVT.

Our findings suggest that supine-positioned LPB is a safe, effective, and straightforward peripheral nerve block technique. This approach eliminates the need for positional changes, thereby increasing patient comfort. Thus, the supine-positioned LPB may emerge as the preferred method for performing ultrasound-guided LPB. However, this study has some limitations. Despite the absence of renal, ureteral, or gut injuries in this study, the close proximity between the needle trajectory and the abdominal organs may lead to organ injury. Therefore, real-time ultrasound monitoring of needle tip location during puncture is necessary to avoid organ damage. Additionally, our investigation failed to compare the efficacy and possible complications of supine-positioned LPB with traditional ultrasound-guided LPB techniques. However, we cannot confirm the superiority of this novel technique over conventional approaches. A prospective randomised controlled study will be conducted to compare the effects and complications of this novel technique with those of traditional ultrasound-guided LPB techniques. Furthermore, the application of supine LPB in other lower extremity surgeries, such as total hip arthroplasty, will be investigated in the future.

In conclusion, the novel ultrasound-guided LPB is a viable technique for peripheral nerve block, offering a high rate of successful blocking outcomes and overall safety. Notably, this novel single-injection LPB combined with SNB affords inferior analgesia after TKA compared with single-injection FNB combined with SNB. However, the novel LPB, in conjunction with SNB, facilitated quicker motor recovery following TKA.
